# Supplementary material for: A Bayesian model of acquisition and clearance of bacterial colonization incorporating within-host variation
Source: PLoS Comput Biol. 2019 Apr 22;15(4):e1006534. doi: 10.1371/journal.pcbi.1006534 (PMC6497309; doi:10.1371/journal.pcbi.1006534)
Supplement: S1 File — We provide some further derivations and details related to our MCMC algorithm. To guide the selection of prior hyperparameters, we also derive the explicit prior distribution and some of its summaries for the parameter t0 and the mean and variance for the prior predictive distribution for the distance. We show some further ABC inference results and also describe further details on computing the acquisition and clearance rates. (PDF) [file pcbi.1006534.s001.pdf]

A Bayesian model of acquisition and clearance of bacterial colonization incorporating within-host variation - Supplementary Text S1

Marko Järvenpää, Mohamad R. Abdul Sater, Georgia Lagoudas, Paul C. Blainey, Loren G. Miller, James A. McKinnell, Susan S. Huang, Yonatan H. Grad, Pekka Marttinen

Contents

1 Derivations and further details of the model **1**
- 1.1 Gibbs sampler step for $(n_{\text{eff}}, \mu, \lambda)$ . . . 1
- 1.2 Gibbs sampler step for η_i . . . 2
- 1.3 Prior distribution for $\mathbf{t}_0$ . . . 3
- 1.4 Prior predictive distribution . . . 4

2 Additional results from ABC inference **5**

3 Details on computing the acquisition and clearance rates **6**

1 Derivations and further details of the model

1.1 Gibbs sampler step for $(n_{\text{eff}}, \mu, \lambda)$

We justify that the sampling steps for the parameters $(n_{\text{eff}}, \mu, \lambda)$ used in the main text will produce a valid Gibbs sampler that targets the correct posterior (in fact, a grid-based approximation of it because the p_{sim} is computed in a grid). Suppose that we are sampling from the joint probability distribution $p(\mathbf{a}, \mathbf{b}, \mathbf{c})$ and $\mathbf{a}, \mathbf{b}$ and $\mathbf{c}$ are random vectors. A standard (systematic scan) Gibbs sampler would repeat the following steps: 1. sample $\mathbf{a}^{(i)} \sim p(\mathbf{a} \,|\, \mathbf{b}^{(i-1)}, \mathbf{c}^{(i-1)})$, 2. sample $\mathbf{b}^{(i)} \sim p(\mathbf{b} \,|\, \mathbf{a}^{(i)}, \mathbf{c}^{(i-1)})$, 3. sample $\mathbf{c}^{(i)} \sim p(\mathbf{c} \,|\, \mathbf{a}^{(i)}, \mathbf{b}^{(i)})$. In the case of our model, we have $\mathbf{a} = (n_{\text{eff}}, \mu)$, $\mathbf{b} = \lambda$ and $\mathbf{c} = (\boldsymbol{\omega}, \mathbf{z}, \boldsymbol{\eta})$ (except that, in the place of step 3, we in fact have additional Gibbs sampling steps from the components of $\mathbf{c}$ and everything is conditioned on the data). Alternatively, we could include $\mathbf{a}$ and $\mathbf{b}$ to the same block of parameters and replace steps 1 and 2 with: 1'. sample $\mathbf{a}^{(i)} \sim p(\mathbf{a} \,|\, \mathbf{c}^{(i-1)}) = \int p(\mathbf{a}, \mathbf{b} \,|\, , \mathbf{c}^{(i-1)}) \,\mathrm{d}\mathbf{b}$, 2'. sample $\mathbf{b}^{(i)} \sim p(\mathbf{b} \,|\, \mathbf{a}^{(i)}, \mathbf{c}^{(i-1)})$. This is equivalent to sampling directly from $(\mathbf{a}^{(i)}, \mathbf{b}^{(i)}) \sim p(\mathbf{a}, \mathbf{b} \,|\, \mathbf{c}^{(i-1)})$.

Consider now the case where we do not sample exactly from $p(\mathbf{a} \,|\, \mathbf{c})$ but use the Metropolis-Hastings algorithm with proposal density $q(\mathbf{a}^* \,|\, \mathbf{a})$ and then sample $\mathbf{b}^* \sim p(\mathbf{b} \,|\, \mathbf{a}^*, \mathbf{c})$, where $\mathbf{a}^*$ is the proposed value from q. The proposal distribution r for this joint sampling is

$$r((\mathbf{a}^*, \mathbf{b}^*) \,|\, (\mathbf{a}, \mathbf{b})) = q(\mathbf{a}^* \,|\, \mathbf{a}) p(\mathbf{b}^* \,|\, \mathbf{a}^*, \mathbf{c}), \tag{1}$$

where $(\mathbf{a}^*, \mathbf{b}^*)$ is the proposed point and $(\mathbf{a}, \mathbf{b})$ the point from the previous iteration. The acceptance probability is then

$$\begin{aligned}
\rho((\mathbf{a}, \mathbf{b}), (\mathbf{a}^*, \mathbf{b}^*)) &= \min\left\{1, \frac{p(\mathbf{a}^*, \mathbf{b}^* \,|\, \mathbf{c})}{p(\mathbf{a}, \mathbf{b} \,|\, \mathbf{c})} \frac{r((\mathbf{a}, \mathbf{b}) \,|\, (\mathbf{a}^*, \mathbf{b}^*))}{r((\mathbf{a}^*, \mathbf{b}^*) \,|\, (\mathbf{a}, \mathbf{b}))}\right\} &(2)\\
&= \min\left\{1, \frac{p(\mathbf{a}^*, \mathbf{b}^* \,|\, \mathbf{c})}{p(\mathbf{a}, \mathbf{b} \,|\, \mathbf{c})} \frac{q(\mathbf{a} \,|\, \mathbf{a}^*) p(\mathbf{b} \,|\, \mathbf{a}, \mathbf{c})}{q(\mathbf{a}^* \,|\, \mathbf{a}) p(\mathbf{b}^* \,|\, \mathbf{a}^*, \mathbf{c})}\right\} &(3)\\
&= \min\left\{1, \frac{p(\mathbf{a}^*, \mathbf{b}^* \,|\, \mathbf{c}) p(\mathbf{b} \,|\, \mathbf{a}, \mathbf{c})}{p(\mathbf{a}, \mathbf{b} \,|\, \mathbf{c}) p(\mathbf{b}^* \,|\, \mathbf{a}^*, \mathbf{c})} \frac{p(\mathbf{a} \,|\, \mathbf{c}) p(\mathbf{a}^* \,|\, \mathbf{c})}{p(\mathbf{a}^* \,|\, \mathbf{c}) p(\mathbf{a} \,|\, \mathbf{c})} \frac{q(\mathbf{a} \,|\, \mathbf{a}^*)}{q(\mathbf{a}^* \,|\, \mathbf{a})}\right\} &(4)
\end{aligned}$$

$$= \min\left\{1, \frac{p(\mathbf{a}^* \mid \mathbf{c})}{p(\mathbf{a} \mid \mathbf{c})} \frac{q(\mathbf{a} \mid \mathbf{a}^*)}{q(\mathbf{a}^* \mid \mathbf{a})}\right\}. \tag{5}$$

This shows that the acceptance probability does not depend on realisation of $\mathbf{b}^*$ (i.e. the sampled λ) and that the sampling procedure of $(n_{\text{eff}}, \mu, \lambda)$ used in the main text is equivalent to performing one blocked Metropolis-Hastings sampling with the proposal r in Eq 1. This ensures that the full algorithm is a valid Gibbs sampler. (In fact, similar arguments are used to show that Gibbs sampling algorithm is generally a special case of Metropolis-Hastings, see Theorem 10.13 in Monte Carlo Statistical Methods by Robert and Casella 2004.)

Empirical experiments showed that this sampling approach produces chains with better mixing compared to other possible approaches such as the straightforward implementation where (n_{eff}, μ) and λ are sampled separately. The algorithm might be valid even if a new λ is sampled also when the proposed (n_{eff}, μ) is rejected but we do not investigate this here.

1.2 Gibbs sampler step for η_i

We derive the conditional density for the parameter $\eta_i, i = 1, \ldots, N$. First of all, neglecting all the terms in the unnormalised posterior that do not depend on current η_i shows that

$$p(\eta_i \mid \mu, \mathbf{z}_i, \lambda, D) \propto (2\eta_i + \mu t_i)^{z_{i2} d_i} \eta_i^{k-1} e^{-(2z_{i2} + \lambda/\mu)\eta_i} \mathbb{1}_{\eta_i \geq 0}, \tag{6}$$

from which we further see that

$$p(\eta_i \mid \mu, \mathbf{z}_i, \lambda, D) \begin{cases} = \text{Gamma}(\eta_i \mid k, \lambda), & \text{if } z_{i2} = 0, \\ = \text{Gamma}(\eta_i \mid k, 2 + \lambda/\mu), & \text{if } z_{i2} = 1 \text{ and } d_i = 0, \\ \propto (2\eta_i + \mu t_i)^{d_i} \eta_i^{k-1} e^{-(2+\lambda/\mu)\eta_i} \mathbb{1}_{\eta_i \geq 0}, & \text{if } z_{i2} = 1 \text{ and } d_i > 0. \end{cases} \tag{7}$$

We show that the conditional density in the last case ($z_{i2} = 1$ and $d_i > 0$) is a finite Gamma mixture. Because d_i is an integer, we use binomial formula and some algebra to obtain

$$\begin{aligned} p(\eta_i \mid \mu, \mathbf{z}_i, \lambda, D) &= c(2\eta_i + \mu t_i)^{d_i} \eta_i^{k-1} e^{-(2+\lambda/\mu)\eta_i} \mathbb{1}_{\eta_i \geq 0} && (8) \\ &= c \sum_{j=0}^{d_i} \binom{d_i}{j} (2\eta_i)^j (\mu t_i)^{d_i - j} \eta_i^{k-1} e^{-(2+\lambda/\mu)\eta_i} \mathbb{1}_{\eta_i \geq 0} && (9) \\ &= c d_i! \sum_{j=0}^{d_i} \frac{2^j (\mu t_i)^{d_i - j} \eta_i^{k+j-1} e^{-(2+\lambda/\mu)\eta_i}}{j!(d_i - j)!} \mathbb{1}_{\eta_i \geq 0} && (10) \\ &= c d_i! \sum_{j=0}^{d_i} \frac{2^j (\mu t_i)^{d_i - j} \Gamma(k+j)}{j!(d_i - j)!(2 + \lambda/\mu)^{k+j}} \text{Gamma}(\eta_i \mid k+j, 2 + \lambda/\mu), && (11) \end{aligned}$$

where c is a normalisation constant and $\Gamma(\cdot)$ is the Gamma function. The weights

$$w_j = \frac{c d_i! 2^j (\mu t_i)^{d_i - j} \Gamma(k+j)}{(2 + \lambda/\mu)^{k+j} j!(d_i - j)!}, \quad j = 0, 1, \ldots, d_i, \tag{12}$$

are clearly positive and must sum to 1 since the formula must be a valid pdf and thus $c = (\sum_{j=0}^{d_i} \tilde{w}_j)^{-1}$, where $\tilde{w}_j$ is an unnormalised weight that is obtained from Eq 12 when $c = 1$. The above weight formula also clearly holds if $d_i = 0$ and then $w_0 = 1$. There appears to be no simple formula for normalisation constant c for general k, however, we do not actually need to normalise the weights at all if we proceed as discussed in the following paragraph.

We now develop a convenient algorithm to sample from the conditional density of η_i. From Eq 12 we see that $w_j / w_{j-1} = (k + j - 1)(d_i - j + 1)/((\mu + \lambda/2) j t_i)$ for $j = 1, \ldots, d_i$. We can thus define unnormalised log-weights, denoted by $\log(\tilde{w}_j)$, which can be computed recursively using the formulas

$$\log(\tilde{w}_0) = \log(1) = 0, \tag{13}$$

$$\log(\tilde{w}_j) = \log(\tilde{w}_{j-1}) + \log\left(\frac{(k+j-1)(d_i - j + 1)}{j}\right) - \log((\mu + \lambda/2)t_i), \quad j = 1, 2, \ldots, d_i. \tag{14}$$

We then use the fact that if $a_1, \dots, a_n$ are logarithms of unnormalised bin probabilities of a Categorical distribution, and if random variables $g_1, \dots, g_n$ follow independently the standard Gumbel distribution that has the cdf $F(g) = \exp(-\exp(-g))$, then $\arg\max_{j=1,\dots,n}\{g_j + a_j\}$ has the Categorical distribution with bin probabilities $\exp(a_j)/\sum_{k=1}^{n}\exp(a_k), j = 1, \dots, n$. We use this fact also to generate random numbers from the conditional density of $\mathbf{z}_i$.

Sampling from the conditional density of η_i can be now done conveniently using the Algorithm 1, where $\tilde{w}_j$ are the unnormalised weights. This algorithm is fast (unless d_i is large) as it does not require numerical root finding, evaluating special mathematical functions or integration unlike standard methods such as e.g. inverse transform sampling which could be alternatively used.

Algorithm 1 Sampling from the conditional density of η_i

```
if z_i2 = 0 then
    return a sample from Gamma(η_i | k, λ/μ)
else
    sample u_0, u_1, ..., u_{d_i} ~ U([0,1])
    compute g_j = −log(−log(u_j)) for j = 0, 1, ..., d_i
    compute l = argmax_{j=0,...,d_i} {g_j + log(w̃_j)}
    return a sample from Gamma(l, 2 + λ/μ)
end if
```

1.3 Prior distribution for $\mathbf{t}_0$

We specified the prior density for $\mathbf{t}_0$ using hierarchical modelling to be able to learn about these values. However, to select the prior hyperparameters k, α and β to reflect our prior knowledge about reasonable values of t_{0i}, we need to obtain the probability law for $\mathbf{t}_0$. The joint prior pdf of $\mathbf{t}_0$ can be obtained by marginalising λ:

$$
\begin{aligned}
p(\mathbf{t}_0) &= \int_0^\infty p(\mathbf{t}_0 \,|\, k, \lambda)\, p(\lambda)\, \mathrm{d}\lambda && (15)\\
&= \int_0^\infty \prod_{i=1}^{N} p(t_{0i} \,|\, k, \lambda)\, p(\lambda)\, \mathrm{d}\lambda && (16)\\
&= \int_0^\infty \prod_{i=1}^{N} \left(\frac{\lambda^k t_{0i}^{k-1} e^{-\lambda t_{0i}}}{\Gamma(k)} \right) \frac{\beta^\alpha}{\Gamma(\alpha)} \lambda^{\alpha-1} e^{-\beta\lambda}\, \mathrm{d}\lambda \mathbb{1}_{\mathbf{t}_0 \geq 0} && (17)\\
&= \int_0^\infty \frac{\beta^\alpha (\prod_{i=1}^{N} t_{0i})^{k-1}}{\Gamma(\alpha)(\Gamma(k))^N} \lambda^{Nk+\alpha-1} e^{-(\beta + \sum_{i=1}^{N} t_{0i})\lambda}\, \mathrm{d}\lambda \mathbb{1}_{\mathbf{t}_0 \geq 0} && (18)\\
&= \frac{\beta^\alpha (\prod_{i=1}^{N} t_{0i})^{k-1}}{\Gamma(\alpha)(\Gamma(k))^N} \frac{\Gamma(Nk+\alpha)}{(\beta + \sum_{i=1}^{N} t_{0i})^{Nk+\alpha}} \mathbb{1}_{\mathbf{t}_0 \geq 0} && (19)\\
&= \frac{\beta^\alpha (\prod_{i=1}^{N} t_{0i})^{k-1}}{B((\alpha, k\mathbf{1}_N))(\beta + \sum_{i=1}^{N} t_{0i})^{Nk+\alpha}} \mathbb{1}_{\mathbf{t}_0 \geq 0}, && (20)
\end{aligned}
$$

where $\mathbf{1}_N$ is an N-dimensional vector with all elements equal to 1 and $B(\cdot)$ denotes the multivariate Beta function. Since

$$
\begin{aligned}
p(t_{0i}) &= \int_{\mathbb{R}_+^{N-1}} \int_0^\infty \prod_{j=1}^{N} p(t_{0j} \,|\, k, \lambda)\, p(\lambda)\, \mathrm{d}\lambda\, \mathrm{d}\mathbf{t}_{j:j\neq i} && (21)\\
&= \int_0^\infty \int_{\mathbb{R}_+^{N-1}} \prod_{j=1}^{N} p(t_{0j} \,|\, k, \lambda)\, \mathrm{d}\mathbf{t}_{j:j\neq i}\, p(\lambda)\, \mathrm{d}\lambda && (22)\\
&= \int_0^\infty p(t_{0i} \,|\, k, \lambda)\, p(\lambda)\, \mathrm{d}\lambda, && (23)
\end{aligned}
$$

the marginals can be obtained from Eq 20 by setting $N = 1$ and it is seen that the marginals are compound Gamma distributions. Specifically, the mean and the variance of t_{0i} are obtained as

$$\mathbb{E}(t_{0i}) = \frac{\beta k}{\alpha - 1}, (\text{if } \alpha > 1), \quad \mathbb{V}(t_{0i}) = \frac{\beta^2 k(k + \alpha - 1)}{(\alpha - 1)^2(\alpha - 2)}, (\text{if } \alpha > 2). \tag{24}$$

The covariance and correlation coefficient are obtained as follows. Consider t_{0i}, t_{0j} such that $i \neq j$. Then we see that

$$\mathbb{E}(t_{0i}t_{0j}) = \int_0^\infty \int_0^\infty \frac{t_{0i}t_{0j}\beta^\alpha t_{0i}^{k-1} t_{0j}^{k-1}\Gamma(2k + \alpha)}{\Gamma(\alpha)(\Gamma(k))^2(\beta + t_{0i} + t_{0j})^{2k+\alpha}} \, \mathrm{d}t_{0i} \, \mathrm{d}t_{0j} \tag{25}$$

$$= \frac{\beta^\alpha \Gamma(2k + \alpha)}{\Gamma(\alpha)(\Gamma(k))^2} \underbrace{\int_0^\infty \int_0^\infty \frac{t_{0i}^k t_{0j}^k \Gamma(2k + \alpha)}{(\beta + t_{0i} + t_{0j})^{2k+\alpha}} \, \mathrm{d}t_{0i} \, \mathrm{d}t_{0j}}_{= \frac{\Gamma(\alpha-2)(\Gamma(k+1))^2}{\beta^{\alpha-2}\Gamma(2k+\alpha)}} \tag{26}$$

$$= \frac{k^2\beta^2}{(\alpha - 1)(\alpha - 2)}, (\text{if } \alpha > 2), \tag{27}$$

where we have recognised the double integral on the second line as the unnormalised pdf in Eq 20 (but with $N = 2$, $k + 1$ in place of k and $\alpha - 2$ in place of α which also implies that the above integral is finite if $\alpha > 2$) and used the fact that this density integrates to 1. It now follows that

$$\mathrm{cov}(t_{0i}, t_{0j}) = \mathbb{E}(t_{0i}t_{0j}) - \mathbb{E}(t_{0i})^2 = \frac{\beta^2 k^2}{(\alpha - 1)^2(\alpha - 2)}, \tag{28}$$

$$\mathrm{corr}(t_{0i}, t_{0j}) = \frac{\mathrm{cov}(t_{0i}, t_{0j})}{\mathbb{V}(t_{0i})} = \frac{1}{k + \alpha - 1}, \tag{29}$$

which hold for $\alpha > 2$.

If $k \geq 1$, then the mode of this density is

$$\mathrm{mode}(\mathbf{t}_0) = \frac{\beta(k - 1)}{\alpha + 1}\mathbf{1}_N. \tag{30}$$

The derivation of this fact goes as follows. If $k \in (0, 1)$, then the density is infinite at $\mathbf{t}_0 = \mathbf{0}$ and the mode does not exist. If $k = 1$, then the mode is clearly $\mathbf{t}_0 = \mathbf{0}$ and it is unique. In the case $k > 1$ the mode can be found by differentiating the logarithm of the density. We obtain

$$\frac{\partial \log p(\mathbf{t}_0)}{\partial t_{0j}} = \frac{k - 1}{t_{0j}} - \frac{Nk + \alpha}{\beta + \sum_{i=1}^N t_{0i}}, j = 1, \ldots, N. \tag{31}$$

Finding the zero of the gradient shows that for all $j = 1, \ldots, N$

$$(Nk + \alpha)t_{0j} - (k - 1)\sum_{i=1}^N t_{0i} = (k - 1)\beta. \tag{32}$$

Symmetry suggests that the solution is the same for all elements j and, clearly, the point $\mathbf{t}_0 = \beta(k - 1)/(\alpha + 1)\mathbf{1}_N$ satisfies these equations. To confirm that this is the only solution, we write the equations as a linear system $((Nk+\alpha)\mathrm{I}_N - (k-1)\mathbb{I}_N)\mathbf{t}_0 = (k-1)\beta\mathbf{1}_N$, where I_N is the $N \times N$ identity matrix and $\mathbb{I}_N$ is a $N \times N$ matrix with all elements equal to 1. Now, each row sum (with diagonal element excluded) is $(N-1)(k-1) < (N-1)k+\alpha+1 = (Nk+\alpha)-(k-1)$ so the coefficient matrix is diagonally dominant and thus non-singular. If $k > 1$ then the density is clearly zero at the boundaries where $t_{0i} = 0$ for some i, and so the mode does not lie on the boundary of the support of the density. If $k \geq 1$, the mode is thus unique and is obtained from Eq 30.

1.4 Prior predictive distribution

We can analytically derive the mean and variance of the prior predictive distribution for a new distance $\tilde{d}$ corresponding to time interval between the sequenced genomes $\tilde{t}$ under the different strain case. These

facts are used to guide the selection of the prior hyperparameters. The full prior density can be visualised using simulation.

Consider the prior $\mu \sim \mathcal{U}([a_\mu, b_\mu])$ and the priors for $\mathbf{t}_0$ and λ as in the main text. The prior predictive mean and variance are then given by

$$\mathbb{E}(\tilde{d}) = l\left(\frac{2\beta k}{\alpha - 1} + \tilde{t}\right), \tag{33}$$

$$\mathbb{V}(\tilde{d}) = \mathbb{E}(\tilde{d}) + \left(\frac{h}{l^2} - 1\right)\mathbb{E}(\tilde{d})^2 + \frac{4h\beta^2 k(k + \alpha - 1)}{(\alpha - 1)^2(\alpha - 2)}, \tag{34}$$

where $l = (a_\mu + b_\mu)/2$ and $h = (a_\mu^2 + a_\mu b_\mu + b_\mu^2)/3$. For Eq 33 we must have $\alpha > 1$ and for Eq 34 $\alpha > 2$.

The derivation of the above facts goes as follows. From Eq 5 of the main text we obtain $\mathbb{E}(\tilde{d} \,|\, \mu, t_{0i}) = \mu(2t_{0i} + \tilde{t})$. Using the law of total expectation shows that $\mathbb{E}(g(\tilde{d})) = \mathbb{E}(\mathbb{E}(g(\tilde{d}) \,|\, \mu, t_{0i}))$. These two fact now imply

$$\mathbb{E}(\tilde{d}) = \mathbb{E}(\mu(2t_{0i} + \tilde{t})) = \mathbb{E}(\mu)(2\mathbb{E}(t_{0i}) + \tilde{t}) = \frac{a_\mu + b_\mu}{2}\left(\frac{2\beta k}{\alpha - 1} + \tilde{t}\right). \tag{35}$$

The derivation of the variance goes similarly. We use a property of Poisson distribution that tells us that $\mathbb{E}(\tilde{d}(\tilde{d} - 1) \,|\, \mu, t_{0i}) = \mu^2(2t_{0i} + \tilde{t})^2$. Now, using the law of total expectation (with $g(\tilde{d}) = \tilde{d}(\tilde{d} - 1)$) and the formulas for the mean and variance of t_{0i}, we obtain

$$\mathbb{E}(\tilde{d}(\tilde{d} - 1)) = \mathbb{E}(\mu^2)\mathbb{E}((2t_{0i} + \tilde{t})^2) = \mathbb{E}(\mu^2)(4(\mathbb{V}(t_{0i}) + \mathbb{E}(t_{0i})^2 + \tilde{t}\mathbb{E}(t_{0i})) + \tilde{t}^2) \tag{36}$$

$$= \frac{a_\mu^2 + a_\mu b_\mu + b_\mu^2}{3}\left(4\left(\frac{\beta^2 k(k + \alpha - 1)}{(\alpha - 1)^2(\alpha - 2)} + \frac{\beta^2 k^2}{(\alpha - 1)^2} + \frac{\beta k\tilde{t}}{\alpha - 1}\right) + \tilde{t}^2\right). \tag{37}$$

The final result now follows by using the fact $\mathbb{V}(\tilde{d}) = \mathbb{E}(\tilde{d}(\tilde{d}-1)) + \mathbb{E}(\tilde{d}) - \mathbb{E}(\tilde{d})^2$ and further simplifications.

2 Additional results from ABC inference

In this short section we show results obtained with an alternative discrepancy function in ABC analysis of the external data D_0. The experiment details are the same as before except that we use the Euclidean distance instead of the L^1 distance in Eq 8 to compare the simulated and observed distributions. The results are shown in Fig A. When the data from the patients A-M is neglected in panel B of Fig A, we see that the resulting posterior is similar as before although with a slightly elevated mutation rate. In the full data case in panel A, the mutation rate is estimated to be even larger, although the shape of the density is still rather similar as before. We believe this happens because, compared to the discrepancy based on L^1 distance, the Euclidean distance gives substantially more weight to those single cases in the data with relatively high values of the observed distances. In the fitting this is compensated by a larger mutation rate. The L^1-based discrepancy is less affected by such outliers. Consequently, we believe that the robust results based on L^1-based discrepancy are more reasonable and thus chose the corresponding ABC posterior as the prior for the mixture model.

3 Details on computing the acquisition and clearance rates

We first describe how to obtain the uncertainty estimates for Table 2 of the main text. We denote the same strain probability in case 1) with $s \in [0, 1]$. The amount of cases in 1) is n and the indicator variable of whether the data point i is of the same strain is $x_i \in \{0, 1\}$ for $i = 1, \ldots, n$. Because the different ST's can be distinguished from each other, n is considered known and fixed. The number of same strain cases $l = \sum_{i=1}^{n} x_i$ is assumed to follow Binomial distribution so that $l \sim \text{Bin}(l \,|\, s, n)$. Using the uninformative prior $s \sim \mathcal{U}([0, 1])$, we then obtain the posterior $p(s \,|\, l) = \text{Beta}(s \,|\, l + 1, n - l + 1)$ which follows from a standard result of Bayesian modelling.

However, we do not know exactly if each data point is of the same strain but only have the posterior probabilities (obtained from the MCMC samples of $p(\mathbf{z} \,|\, D, D_0)$) which complicates the analysis. However,

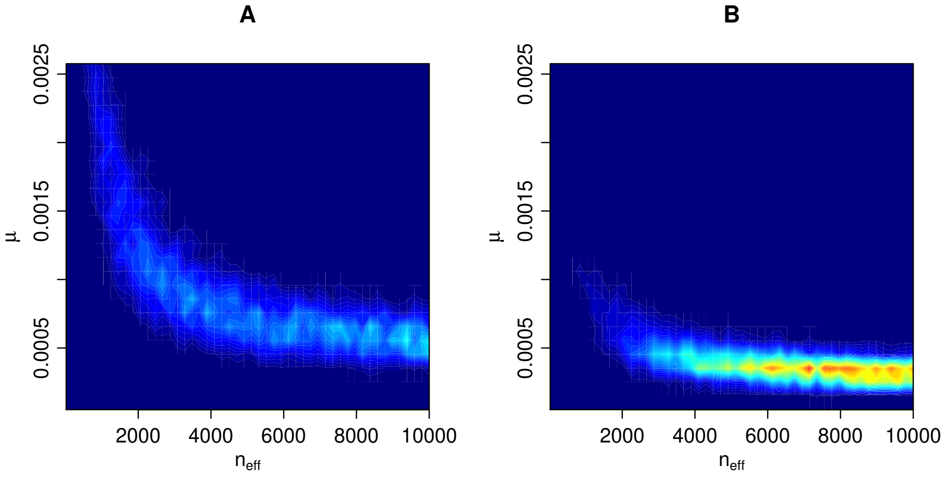

Figure A: **ABC posterior distribution for $(n_{\mathbf{eff}}, \mu)$ with an alternative discrepancy.** Panel A shows the result with the full data and panel B the corresponding result with only a subset of the data.

we see that

$$p(s \mid D, D_0) = \sum_{l=0}^{n} p(l, s \mid D, D_0) = \sum_{l=0}^{n} p(s \mid l) p(l \mid D, D_0), \tag{38}$$

which shows that the posterior of s is a Beta mixture density where the weights $p(l \mid D, D_0)$ are the posterior probabilities for the potential amounts of same strain cases which can be computed from the existing MCMC chain of the mixture model. We immediately obtain $\mathbb{E}(s \mid D, D_0) = \sum_{l=0}^{n} \frac{l+1}{n+2} p(l \mid D, D_0)$ and the credible interval can be computed numerically by inverting the cumulative distribution function corresponding to Eq 38. The above computations similarly apply to the other cases as 1).

As a final part of this section, we describe in detail how the acquisition and clearance rates and their uncertainty estimates in Table 3 of the main text are computed. If the samples are consecutive and not negative, then the definitions A-C of Table 3 are used directly. If negative samples are involved, then we must deal with potential "false negatives". Because we cannot know for sure if each negative sample is false negative (or, generally, what is exactly happening between any pair of samples) and to keep our analysis here simple enough, we need to make some strict assumptions. Table A contains related rules that are used to determine the acquisition and clearance events in the cases that involve negative samples. For example, the first line of Table A shows the case where the last sample is negative while the preceding sample is not. We assume that in this case we always have one clearance event (i.e. D in Table 3) although it would be also possible that this is false negative and the patient was in fact still colonised. Additionally, for the events on lines 3-6 and depending on the posterior probability of the same strain corresponding to each case, we assume either that the strain is not decolonised (i.e. each negative sample is false negative) or that there has been one clearance and one acquisition event.

To take into account the posterior uncertainty in the same and different strain classifications, we compute the quantities A-E for each parameter sampled from the posterior and then compute the acquisition and clearance rates for each of these values. (In fact, unlike other values, C is not random since ST's can be determined for sure.) Finally, the mean and the 95% quantile are computed numerically from the resulting set of samples of acquisition and clearance rate values.

Table A: **Definition of cases with negative sample(s) involved.**

ST A, str X $\rightarrow \emptyset$ (END)	one clearance D
ST A, str X $\rightarrow \emptyset \rightarrow ... \rightarrow \emptyset$ (END)	one clearance D
ST A, str X $\rightarrow \emptyset \rightarrow$ ST A, str X	two events A (prob)
ST A, str X $\rightarrow \emptyset \rightarrow$ ST A, str Y	one D + one E (prob)
ST A, str X $\rightarrow \emptyset \rightarrow ... \rightarrow \emptyset \rightarrow$ ST A, str X	multiple events A (prob)
ST A, str X $\rightarrow \emptyset \rightarrow ... \rightarrow \emptyset \rightarrow$ ST A, str Y	one D + one E (prob)
ST A $\rightarrow \emptyset \rightarrow$ ST B	one D + one E
ST A $\rightarrow \emptyset \rightarrow ... \rightarrow \emptyset \rightarrow$ ST B	one D + one E

(END) illustrates the final visit to hospital (i.e. visit V4) and (prob) emphasises that the posterior probability of the same strain is used for determining whether clearance or other event could have happened.
